# A Bioclinical Pattern for the Early Diagnosis of Cardioembolic Stroke

**DOI:** 10.1155/2014/242171

**Published:** 2014-03-05

**Authors:** Bruno Zecca, Clara Mandelli, Alberto Maino, Chiara Casiraghi, Giovanbattista Bolla, Dario Consonni, Paola Santalucia, Giuseppe Torgano

**Affiliations:** ^1^Emergency Care Unit, Fondazione IRCCS Ca' Granda OspedaleMaggiore Policlinico, Via F. Sforza 35, 20100 Milan, Italy; ^2^Università degli Studi di Milano, Via Festa del Perdono 7, 20100 Milan, Italy; ^3^Angelo Bianchi Bonomi Hemophilia and Thrombosis Centre, Fondazione IRCCS Ca' Granda Ospedale Maggiore Policlinico, Via F. Sforza 35, 20100 Milan, Italy; ^4^Cardiovascular Disease Institute, Fondazione IRCCS Ca' Granda Ospedale Maggiore Policlinico, Via F. Sforza 35, 20100 Milan, Italy; ^5^Epidemiology Unit, Fondazione IRCCS Ca' Granda Ospedale Maggiore Policlinico, Via F. Sforza 35, 20100 Milan, Italy; ^6^Scientific Direction, Fondazione IRCCS Ca' Granda Ospedale Maggiore Policlinico, Via F. Sforza 35, 20100 Milan, Italy

## Abstract

*Background and Scope*. Early etiologic diagnosis of ischemic stroke subtype guides acute management and treatment. We aim to evaluate if plasma biomarkers can predict stroke subtypes in the early phase from stroke onset. *Methods*. Plasma N-terminal prohormone of brain natriuretic peptide (NT-proBNP), D-dimer, C-reactive protein, serum albumin, and globulin levels have been investigated in 114 consecutive patients presenting at the emergency room within 6 hours of the ischemic stroke onset. Plasma levels of biomarkers have been correlated with stroke aetiology (based on TOAST criteria) by multivariable logistic regression analysis, adjusted for several covariates. *Results*. Of the 114 patients, 34 (30%) had cardioembolic stroke, 27 (23%) atherothrombotic stroke, 19 (17%) lacunar stroke, and 34 (30%) stroke of undetermined origin. Patients with cardioembolic stroke had significantly higher levels of NT-proBNP and lower globulin/albumin (G/A) ratio compared with the other subgroups. At multiple logistic regression NT-proBNP > 200 pg/mL, G/A ratio > 0.70, and NIHSS score were independent predictors of cardioembolic stroke with high accuracy of the model, either including (AUC, 0.91) or excluding (AUC, 0.84) atrial fibrillation. *Conclusions*. A prediction model that includes NT-proBNP, G/A ratio, and NIHSS score can be useful for the early etiologic diagnosis of ischemic stroke.

## 1. Introduction

Etiological classification of acute ischemic stroke is based on probabilistic criteria, defined by TOAST [1]. Nearly one-third of ischemic strokes are classified as undetermined, including those with two or more well-identified causes. A correct assessment of aetiology is crucial for management in the acute and subacute phases, in order to give the patient the prompt treatment and the best prevention of early recurrence.

It has been suggested that biological markers could be used in etiologic identification of ischemic stroke. For example, in stroke of cardioembolic origin, levels of brain natriuretic peptide (BNP), known to be related to severity and mortality [2–7], have been shown to be higher than other stroke subtypes [8, 9]. Moreover, some authors identified cut-off values above which the predictive value for cardioembolic stroke (CES) is very high [8–12].

The predictive value of BNP in CES is even increased when associated with markers of thromboembolism and heart disease, as reported by Montaner et al. [10]. In patients with total anterior circulation syndrome, they found that a BNP level greater than 76 pg/mL combined with a D-dimer (DD) level greater than 0.96 mcg/mL predicts nearly 90% of all CES. The authors concluded that the identification of a third biomarker would further increase the diagnostic accuracy.

It is widely reported that low albumin level correlates with an increased risk of cardiovascular diseases and atrial fibrillation (AF) [13–16]. Emerging evidence suggests a tight relationship between some inflammatory patterns (i.e., high fibrinogen, C-reactive protein, low albumin, and albumin/globulin ratio) and risk of CES in patients with nonvalvular atrial fibrillation [17–19].

The aim of this study is to evaluate the predictive value of a panel of specific biomarkers, easy feasible in the emergency setting, in the early assessment of stroke aetiology.

## 2. Methods

### 2.1. Patients

From December 2003 to June 2008 consecutive patients with acute ischemic stroke, admitted to the emergency department of our hospital within 6 hours from stroke onset, were included in this study. Detailed inclusion criteria were age of 18–90 years, presentation within 6 hours of the onset of acute ischemic stroke, symptoms lasting more than 60 minutes, NIHSS score from 5 to 25, and absence of cerebral haemorrhage at the baseline CT scan.

At the emergency department all patients underwent thorough evaluation including clinical history (present or past history of hypertension, atrial fibrillation, diabetes, hyperlipidaemia, and smoking), physical and neurological examination, and a first panel of tests such as complete blood work, electrocardiogram (ECG), noninvasive arterial blood pressure monitoring, chest radiography, and unenhanced CT scan and/or angio-CT. During the following hospital stay all patients were further investigated with a second panel of tests including carotid and cardiac ultrasound (transthoracic and, in case, transesophageal) and, in selected cases, MRI-MRA, cerebral angiography, transcranial duplex (TCD), and 24 h ECG monitoring.

### 2.2. Blood Biomarkers Sampling

Blood samples for specific biomarkers determination have been collected at inclusion in the emergency department. Samples were centrifuged at 3000 rpm for 15 minutes, and plasma was frozen and stored at the temperature of −20°C. Ultrasensitive C-reactive protein (CRP), D-dimer, albumin, and globulins were assayed with an automated latex enhanced immunoassay or colorimetric assay with endpoint method. Plasma N-terminal prohormone of brain natriuretic peptide (NT-proBNP) level was measured using a RIA (Human RIA Kit Phoenix Pharmaceuticals).

### 2.3. Stroke Classifications

Stroke severity was assessed by the National Institutes of Health Stroke Scale (NIHSS) [20]. At discharge, stroke etiologic subtypes were identified according to the TOAST classification (Trial of Org 10172 in Acute Stroke Treatment) [1], which distinguishes five stroke categories: large-artery atherosclerosis or atherothrombotic, cardioembolic, small-vessel occlusion or lacunar, undetermined or cryptogenic, and other determined aetiologies (rare causes of stroke). To determine the TOAST category all the information available from the hospital admission was taken into account, including the panel of tests performed during the hospital stay.

Stroke was also classified according to the Oxfordshire Community Stroke Project criteria (OCSP) [21], based on clinical symptoms, location, and extent of cerebral infarction. OCSP classification distinguishes strokes as total anterior circulation infarcts (TACI), partial anterior circulation infarcts (PACI), lacunar infarcts (LACI), and posterior circulation infarcts (POCI). Mortality was assessed at three months.

### 2.4. Statistical Analysis

For descriptive statistics we used median for nominal and ordinal variables (such as stroke scales) and mean and standard deviation (SD) for continuous variables (i.e., NT-proBNP values). Differences among groups were assessed by chi-square test for categorical variables and Kruskal-Wallis test for continuous variables.

The optimal cut-off points of plasma NT-proBNP, D-dimer, CRP, albumin, plasma globulin, and globulin/albumin ratio to identify CES versus non-CES were obtained using the receiver operating characteristic (ROC) curves. For selected biomarkers sensitivity and specificity for the diagnosis of CES and their 95% Agresti-Coull confidence intervals were calculated after the exclusion of patients with undetermined causes. All variables were tested one by one against the dependent variable CES; variables associated with CES at univariate analysis were entered into a logistic regression model and odds ratios (OR) with their 95% confidence interval (CI) were calculated. Biomarkers were included as dichotomous variables (0 = less than or 1 = greater than the cut-off value), vascular territory and etiologic subtype as nominal variable, and NIHSS score and age as continuous variables. Goodness of fit of the multiple logistic model was tested with the Hosmer-Lemeshow test. An equation from the regression model was derived to calculate the probability of cardioembolic aetiology. Statistical analyses were performed using the Stata software, version 11.

## 3. Results

During the study period 114 patients fulfilled the inclusion criteria and were included in this study. Of those, at the end of the hospital stay, 34 (30%) were classified as having CES, 27 (23%) having stroke of large-artery atherosclerosis, and 19 (17%) having stroke of small artery occlusion or lacunae. In 34 of 114 patients (30%) the diagnostic workup was inconclusive (27 patients) or there was coexistence of two or more potential causes (7 patients, including 3 patients with AF at the emergency department and subsequent evidence of ipsilateral symptomatic stenosis) and therefore were classified as having stroke of undetermined cause. No one had stroke of other determined causes.

Mean age was 71.7 (SD 12.1) years. The distribution of demographic and vascular risk factors was similar among the TOAST groups, except for a history of diabetes which was more frequent in patients with large vessels atherosclerosis (41% versus 6–12% in the other groups) and for the presence of AF at the emergency room in patients with CES (82% versus 10–16% in the other groups). Patients with CES were more severely affected than all the other groups (median NIHSS score at presentation 14 versus 6–9) and had a higher short term mortality rate (Table 1). Stroke subtypes according to TOAST and Oxfordshire Community Stroke Project classifications are reported in Table 1.

### 3.1. Biomarkers

Baseline blood samples were collected at a median time from stroke onset of 240 minutes (Q1 41–Q3 340 minutes). As shown in Table 2, the highest NT-proBNP value was observed in CES, and the lowest in patients with lacunar stroke. Albumin level was higher among patients with large-artery atherosclerosis than other groups, and the G/A ratio was higher in CES than other groups. Biomarkers that showed a significant association with CES were NT-proBNP and G/A ratio.

For these biomarkers, the optimal cut-off value for discriminating the presence of a cardioembolic source was a level of NT-proBNP of 200 pg/mL and a ratio of G/A of 0.7. Using this cut-off values we calculate sensitivity and specificity of 0.65 (95% CI 0.48–0.79) and 0.82 (95% CI 0.73–0.89) for NT-proBNP and 0.91 (95% CI 0.76–0.98) and 0.31 (95% CI 0.22–0.42) for G/A ratio.

### 3.2. Bioclinical Model for CES

**Figure 1:**
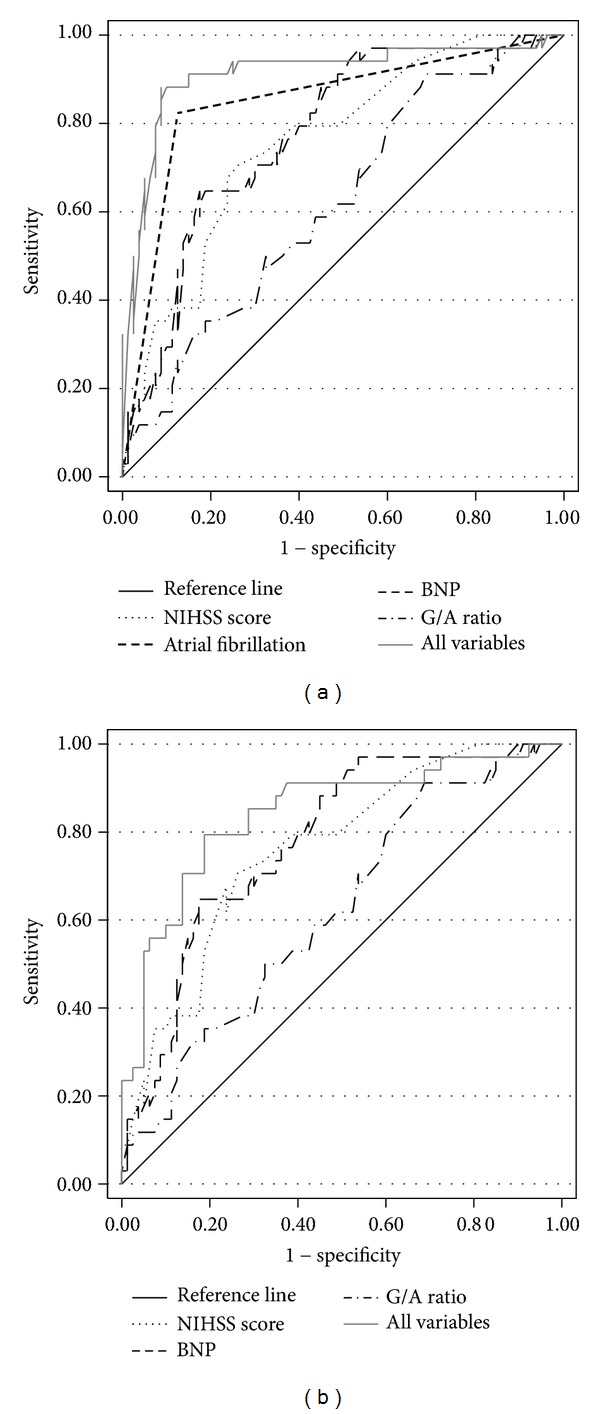
Receiver operating characteristic (ROC) curves for selected clinical variables among 114 patients (34 with cardioembolic stroke and 80 with stroke of other origins), alone or in combination in multiple logistic regression models. (a) Model with atrial fibrillation; (b) model without atrial fibrillation. See Table 3 for model specification.

When biomarkers that showed a significant association with CES (A/G ratio and NT-proBNP) were evaluated in a multivariable logistic regression model including the baseline clinical characteristics, the strongest predictors of a stroke of cardioembolic origin were the presence of atrial fibrillation (OR 24.67, 95% CI 6.46–94.22), NT-proBNP levels above 200 pg/mL (OR 3.96, 95% CI 1.02–15.36), G/A ratio above 0.7 (OR 3.87, 95% CI 0.70–20.98), and NIHSS score (OR for each unit increase 1.10, 95% CI 0.99–1.22) (Table 3). Since in the clinical practice the diagnosis of cardioembolic stroke is strongly related to the presence of AF, we repeated the analysis after excluding it, and results for BNP levels, NIHSS score, and globulin/albumin ratio were even stronger.  Figure 1 shows the ROC curves corresponding to the two regression models with and without AF. The areas under the curve (AUCs) were good for both models (0.91 with AF and 0.84 without AF).

The regression equations obtained were the following:

1. with AF: log(odds) = −1.91 + 0.27 × SEX-0.04 × AGE + 0.09 × NIHSS + 1.38 × NT-proBNP + 1.35 × G/A + 3.21 × AF;
2. without AF: log(odds) = −4.19 + 0.31 × SEX-0.02 × AGE + 0.12 × NIHSS + 1.77 × NT-proBNP + 1.42 × G/A.

The application of model 1 to the undetermined aetiology group allowed the identification of 4 patients (4/34, 12%) with probability of CES between 70 and 90%. Of those, only one patient presented at the emergency department with AF.

## 4. Discussion

We found that NT-proBNP and G/A ratio are good predictors of CES in the early phase of diagnostic workup and they can increase the prediction accuracy for CES of the clinical signs alone, such as AF and event's severity.

An early stroke classification is important for therapeutic choices and for improving outcome. Among the different etiologic subtypes, CES is associated with higher risk of early recurrences; therefore, its prompt identification may improve patient's prognosis. Several studies evaluated laboratory and clinical predictors of CES. A good association was observed between CES and BNP levels [4], and an even stronger predictive value was found when BNP measurement was combined with D-dimer and clinical syndrome of the anterior cerebral circulation [10]. Based on this literature evidence, we tried to define another feasible clinical-biochemical stroke profile, using blood tests promptly available in our emergency laboratory.

BNP is released primarily from cardiac ventricles in response to volume and pressure overload. It could be a biomarker of ventricular dysfunction and AF [22, 23] and its increase could be related to blood stasis, which is a well-known condition for thrombi formation and risk of embolism [24, 25]. The peptide and its terminal fragment NT-proBNP were found elevated in patients with acute ischemic stroke, and their plasma level was associated with stroke severity and mortality [3, 5, 6]; in addition, increased BNP levels are suggestive of a cardioembolic source for the cerebrovascular event [7–10, 12]. In our study, BNP alone showed sensitivity for CES definition of 65% and a positive predictive value of 61%, which is in line with other series reports [25].

It is widely accepted that inflammation represents a risk factor for AF and for prothrombotic conditions [24–27]. Different molecules behave differently during an inflammatory phase; albumin synthesis decreases, while other inflammatory globulins rise (i.e., fibrinogen, CRP, alpha-2-macroglobulin, etc.). This pattern is regulated by circulating cytokines, mostly IL-6. Albumin and globulins variations could also be suggestive of a prothrombotic state [17], and globulin/albumin ratio (G/A) correlates with blood viscosity, being a high ratio associated with increased blood viscosity.

Our results support the hypothesis that specific biomarkers could be a useful tool for early identification of stroke subtypes, mainly CES. We identified a “cardioembolic pattern” that includes stroke severity, BNP > 200 pg/mL and G/A ratio > 0.7, which was a good predictor of CES, reaching an accuracy of nearly 90%. These findings confirm previous literature data [7, 28, 29], supporting the suggestion that the combination of clinical and biochemical variables leads to a better definition of stroke aetiology. Differently from Montaner et al., who identified BNP and D-dimer as good predictors of CES, in our series D-dimer turned out not to be as good as G/A ratio in predicting CES.

Our model is easily applicable in the emergency room and can be useful for the identification of patients with a stroke profile at high risk for early recurrence and unfavourable outcome such as CES.

Limitations of our study are the small number of the patient's series and the slow recruitment rate. All patients were recruited within 6 hours from stroke onset and therefore represent a peculiar stroke patient population. This may have implied a higher proportion of CES compared to other series because CES is usually characterized by abrupt onset causing the patient to reach medical attention more quickly. The high proportion of CES, however, did not affect the results of our analysis. Moreover, in the common practice, etiologic classifications are limited by the lack of a clear cut definition of ischemic stroke in the undetermined category, where patients with cardioembolic causes are also included. To overcome this issue, a new phenotype-based classification, the A-S-C-O system, was developed few years ago [30]. This system, different from the TOAST classification that considers only the most likely cause(s) of stroke, aims to better describe the phenotype of the patients and the aetiology of the stroke, without neglecting the information. However, the recruitment and the data collection took place before the publication of the A-S-C-O stroke classification system; therefore, a direct comparison with the proposed bioclinical model was not possible.

## 5. Conclusions 

NT-proBNP, albumin, and globulin are useful for early diagnosis of CES; the increase of NT-proBNP over 200 pg/mL, G/A ratio > 0.7, with or without AF and in presence of severe stroke, supports the suspicion of cardioembolism. We think that the identification of a specific clinical-biochemical profile could help in the emergency setting to better address the most appropriate instrumental diagnostic workup in selected patients with ischemic stroke.

## Figures and Tables

**Table 1:** Demographic and clinical features in 114 patients with stroke according to TOAST classification.

	Cardioembolic	Large-artery atherosclerosis	Small-vessel occlusion (lacune)	Undetermined aetiology	*P* value*
	*n* = 34	*n* = 27	*n* = 19	*n* = 34	
Demographics					
Females	12 (35%)	15 (56%)	11 (58%)	13 (38%)	0.22
Males	22 (65%)	12 (44%)	8 (42%)	21 (62%)	
Age, mean (SD)	75.9 (8)	74.1 (9)	69.8 (13.7)	71.3 (14)	0.50
History					
Smoking	5 (15%)	7 (26%)	7 (37%)	7 (21%)	0.30
Hypertension	26 (76%)	23 (85%)	15 (79%)	24 (71%)	0.60
Dyslipidaemia	21 (62%)	17 (63%)	10 (52%)	15 (44%)	0.39
Diabetes	4 (12%)	11 (41%)	2 (10%)	2 (6%)	0.002
Previous stroke	8 (23%)	11 (41%)	2 (10%)	4 (12%)	0.03
Event					
Atrial fibrillation at the ER admission	28 (82%)**	4 (15%)	3 (16%)	3 (9%)	<0.001
Left atrial enlargement***	15 (56%)	8 (40%)	3 (19%)	7 (28%)	0.07
Baseline NIHSS score, median (*Q*1–*Q*3)	14.0 (9.0–19.0)	9.0 (6.0–16.0)	6.0 (5.0–7.0)	7.5 (6.0–13.0)	0.0001
3-month mortality	10 (29%)	7 (26%)	1 (5%)	1 (12%)	0.08
Location****					
Lacunar infarct (LACI)	2 (6%)	10 (37%)	18 (95%)	2 (6%)	<0.001
Partial anterior circulation infarct (PACI)	9 (26%)	8 (30%)	1 (5%)	20 (59%)	
Total anterior circulation infarct (TACI)	23 (68%)	8 (30%)	0 (0%)	9 (26%)	
Posterior circulation infarct (POCI)	0 (0%)	1 (3.7)	0 (0%)	3 (9%)	

Abbreviations: *Q*1: first quartile; *Q*3: third quartile; SD: standard deviation; ED: emergency department.

*From chi-square (categorical) or Kruskal-Wallis test (continuous variables).

**Of the six patients with CES without AF at the emergency department, one had an echocardiographic finding of thrombus in the left atrium and five had developed AF during the hospital stay.

***Based on 88 (27, 20, 16, and 25) US echocardiographic examinations.

****According to Oxford Community Stroke Project (OCSP) classifications.

**Table 2:** Biomarker distribution in 114 patients according to TOAST classification.

	Cardioembolic	Large-artery atherosclerosis	Small-vessel occlusion (lacune)	Undetermined aetiology	*P* value*
	*n* = 34	*n* = 27	*n* = 19	*n* = 34	
NT-proBNP (pg/mL)	254 (149–327)	137 (79–176)	86 (40–183)	117 (75–188)	0.0001
Albumin (g/mL)	3.9 (3.8–4.1)	4.1 (3.8–4.2)	4.0 (3.9–4.4)	3.9 (3.7–4.1)	0.04
Globulins (g/mL)	3.2 (2.9–3.4)	2.9 (2.8–3.3)	3.0 (2.6–3.4)	3.0 (2.7–3.3)	0.45
Globulin/albumin	0.79 (0.72–0.88)	0.74 (0.70–0.79)	0.76 (0.62–0.83)	0.77 (0.69–0.85)	0.13
CRP (*μ*g/mL)	0.48 (0.23–0.98)	0.37 (0.14–0.62)	0.43 (0.14–1.48)	0.34 (0.15–1.65)	0.44
DD (*μ*g/mL)**	406 (282–845)	408 (239–724)	304 (185–435)	308 (196–588)	0.17

All values are median (first and third quartile).

*From chi-square (categorical) or Kruskal-Wallis test (continuous variables).

**Determinations made on 101 (31, 23, 17, and 30) US echocardiographic examinations.

**Table 3:** Results of multiple logistic regression model for the likelihood of a stroke of cardioembolic origin among 114 patients. Odds ratios (OR) and 95% confidence intervals (95% CI) are estimated from multiple logistic regression models including and excluding atrial fibrillation (AF).

	Cardioembolic stroke		Model with AF			Model without AF		
	No	Yes	OR*	95% CI	*P* value	OR*	95% CI	*P* value
Sex								
Female	39	12	1.00	Reference		1.00	Reference	
Male	41	22	1.31	0.38–4.54	0.67	1.36	0.47–3.93	0.57
Age (years)	80	34	0.96	0.90–1.02	0.16	1.00	0.95–1.05	0.92
Baseline NIHSS score	80	34	1.10	0.99–1.22	0.09	1.14	1.04–1.24	0.005
NT-proBNP (pg/mL)								
<200	66	12	1.0	Reference		1.0	Reference	
≥200	14	22	3.96	1.02–15.36	0.047	5.90	2.03–17.14	0.01
Globulin/albumin								
<0.70	25	3	1.0	Reference		1.0	Reference	
≥0.70	55	31	3.84	0.70–20.98	0.12	4.13	1.00–17.06	0.05
Atrial fibrillation								
No	70	6	1.00	Reference				
Yes	10	28	24.67	6.46–94.22	<0.001			
Area under ROC curve			0.91			0.84		

*Each variable is adjusted for the others.
